# Manuka honey as a natural remedy for diabetic foot ulcers: efficacy compared with silver-based dressing

**DOI:** 10.3389/fphar.2026.1790277

**Published:** 2026-03-30

**Authors:** Ewa Rojczyk, Kinga Spyrka, Aleksander Sieroń, Dariusz Bazaliński, Marek Kucharzewski

**Affiliations:** 1 Wladyslaw Bieganski Collegium Medicum, Jan Długosz University in Częstochowa, Częstochowa, Poland; 2 Faculty of Medicine, Academy of Silesia in Katowice, Katowice, Poland; 3 Institute of Health Sciences, Collegium Medicum, University of Rzeszów, Rzeszów, Poland; 4 Wound Treatment Clinic of Specialist Hospital of priest B. Markiewicz Subcarpathian oncology Centre in Brzozowo, Brzozowo, Poland; 5 Department of General Surgery, Surgical Outpatient Clinic of Healthcare Centre of Jan Paweł II District Hospital in Włoszczowa, Włoszczowa, Poland

**Keywords:** diabetes, healing, honey, ulcers, wounds

## Abstract

**Introduction:**

Diabetic foot ulcers (DFUs) present significant challenges in clinical management. Numerous studies have confirmed the effectiveness of honey in promoting the healing of cutaneous ulcers of various etiologies, including DFUs. The aim of the study is to compare the effectiveness of DFUs treatment with Manuka honey with the use of a classic silver-based dressing.

**Methods:**

A randomized controlled trial was conducted involving two groups of patients (a total of 60 individuals) with DFUs. Patients were randomly assigned to two groups: the experimental group received treatment with medical-grade Manuka honey, whereas the control group was treated with a silver-containing calcium alginate dressing. The investigated Manuka honey product was reported with full device identifiers to ensure clinical-grade product traceability. RESVECH 2.0 Scoring Table was used twice (just before procedure and after 4 weeks) to assess treatment effectiveness. It is a validated multidimensional scale designed to assess and monitor the progression of chronic wound healing across key clinical parameters.

**Results:**

With the exception of Depth/Tissues Involved, all evaluated parameters of RESVECH 2.0 Scoring Table demonstrated a significantly greater improvement in the experimental group compared with the control group (p < 0.001). Total score was reduced by 41.3% in the experimental group vs. 2.0% in the control group. Statistically significant, mostly positive correlations were found between wounding time and RESVECH 2.0 scores before and after 4 weeks of therapy, as well as between wounding time and the magnitude of treatment effect.

**Discussion:**

Manuka honey demonstrated greater efficacy than silver-based dressings in promoting the healing of diabetic foot ulcers, supporting its use as an effective alternative in clinical practice.

## 1 Introduction

Diabetic foot ulcers (DFUs) affect an estimated 18.6 million individuals globally each year and are strongly correlated with elevated risks of limb amputation and mortality. Standard therapeutic approaches include surgical debridement, offloading to minimize mechanical pressure, management of peripheral ischemia and infection, and prompt referral to a multidisciplinary care team (Armstrong et al., 2023).

Diabetic foot ulceration arises from a multifactorial interplay of neurological, vascular, and biomechanical impairments. It is estimated that 50%–60% of these ulcers develop infections, with approximately 20% of moderate to severe cases progressing to lower limb amputation. The 5-year mortality rate for patients with diabetic foot ulcers is around 30%, increasing to over 70% among those undergoing major amputation. Furthermore, the mortality incidence among individuals with DFUs is 231 per 1000 person-years, in contrast to 182 per 1000 person-years observed in diabetic patients without foot ulceration (Armstrong et al., 2023).

The global burden of diabetes is steadily increasing, with the International Diabetes Federation reporting that, in 2021, approximately 537 million adults aged 20 to 79 were affected by the disease. This growing prevalence contributes to a heightened occurrence of diabetes-related foot complications, particularly infections (IDF Diabetes Atlas 10th Editon, 2021). Diabetic foot infections (DFIs) are linked to significant clinical consequences, often necessitating regular medical consultations, intensive wound management, antibiotic treatment, surgical interventions, and substantial healthcare expenditures (Raspovic and Wukich, 2014). Notably, DFIs constitute the most common diabetes-associated complication requiring hospital admission and represent the leading cause of lower limb amputations among individuals with diabetes (Peters et al., 2001; Lavery et al., 2007).

Numerous guidelines have been developed to support clinicians in the management of DFIs. The Infectious Diseases Society of America (IDSA) initially released a guideline in 2004, which was subsequently revised in 2012 (Lipsky et al., 2004; 2012). Since 2004, the International Working Group on the Diabetic Foot (IWGDF) has published comprehensive guideline documents every 4 years (Peters et al., 2016). The most recent 2023 iteration of the IWGDF guideline represents an update to the 2019 version, focusing on the diagnosis and management of DFIs, and forms part of the broader IWGDF guideline series (Lipsky et al., 2020). For the first time, the IWGDF and IDSA have collaborated to produce a unified intersociety guideline, incorporating contributions from experts affiliated with both organizations in the development of a single, consolidated document. This guideline recommends a multidisciplinary approach to diabetic foot ulcer management, including prompt wound debridement, appropriate off-loading, assessment and optimization of vascular status, targeted antimicrobial therapy when infection is present, and comprehensive metabolic control as the cornerstone of best clinical practice.

The development of DFUs is closely linked to the complex pathophysiological consequences of chronic hyperglycemia, which impairs the normal wound-healing cascade at multiple levels. Diabetic fibroblasts exhibit reduced proliferative activity and altered morphology, contributing to delayed tissue regeneration (Loots et al., 1999). In addition, insufficient leukocyte recruitment and disturbed immune cell signaling have been observed at ulcer margins (Galkowska et al., 2005). Impaired responsiveness of monocytes to angiogenic stimuli such as vascular endothelial growth factor (VEGF) further limits neovascularization and granulation tissue formation (Waltenberger et al., 2000). Keratinocyte migration and differentiation are also disrupted, preventing effective re-epithelialization of chronic wounds (Usui et al., 2008). Moreover, defective mobilization of bone marrow–derived progenitor cells and a predominance of pro-inflammatory macrophage phenotypes exacerbate the persistence of inflammation and hinder proper tissue repair (Albiero et al., 2011; Kanter et al., 2012).

Although various topical dressings are employed by healthcare professionals in an effort to promote healing of DFUs, there is a lack of robust, high-quality evidence to inform standardized clinical decision-making regarding their use (Bergin and Wraight, 2006; Jull et al., 2015).

The utilization of silver for the prevention and treatment of infections and various illnesses can be traced back to approximately 100 BC, when it was employed for such purposes by ancient Greek and Roman civilizations (White, 2001). Interest in silver-based therapeutics was revitalized in the late 19th century, particularly for the management of venereal diseases and ocular infections (Lansdown, 2002). Similarly, honey has a long-standing history of topical application, having been used for centuries by cultures including the Egyptians, Greeks, Romans, and Chinese (Dunford et al., 2000; Jull et al., 2015). Despite its historical prominence, the therapeutic use of honey declined around the 1940s with the emergence and widespread adoption of antibiotics. However, the contemporary rise in antibiotic-resistant bacterial strains—attributed to excessive antibiotic usage—has renewed scientific and clinical interest in honey and silver as alternative or adjunctive therapies for managing chronic wounds, including DFUs, venous leg ulcers (VLUs), and pressure ulcers (Sare, 2008).

Natural honey is primarily composed of sugars and water, with carbohydrates representing over 95% of its dry weight. In addition to its carbohydrate matrix, honey contains more than 200 bioactive compounds, including vitamins, minerals, amino acids, organic acids, flavonoids, and enzymes (Kingsley, 2001; Mandal and Mandal, 2011; Moniruzzaman et al., 2013). Its composition and therapeutic potential are influenced by factors such as floral source, geographic origin, climate, and processing methods. Owing to its complex chemical profile, honey exhibits antioxidant, anti-inflammatory, and strong antibacterial properties. The latter are mainly attributed to its high osmolarity, low pH (3.2–4.5), and the enzymatic production of hydrogen peroxide upon dilution (Molan, 2002; Cutting, 2007). Moreover, honey is non-toxic, non-allergenic, and maintains a moist wound environment conducive to healing. Given its safety, affordability, and antimicrobial efficacy, honey has gained renewed attention as an effective topical agent for wound management (Lusby et al., 2002; Lee et al., 2011; Yaghoobi et al., 2012; Jull et al., 2015; Tashkandi, 2021). However, only honey that has been appropriately sterilized should be considered suitable for therapeutic application.

Indeed, purified, medicinal honey, when utilized as a wound dressing, contributes to maintaining a moist wound environment and exhibits broad-spectrum antimicrobial activity. It demonstrates anti-inflammatory effects, mitigates edema and exudate production, promotes angiogenesis and granulation tissue development, facilitates wound contraction, enhances collagen synthesis, supports autolytic debridement, and accelerates re-epithelialisation of the wound surface (Molan, 1999; Molan, 2006; Sharp, 2009; Al-Waili et al., 2011; Jull et al., 2015). The effectiveness of honey in promoting the healing of cutaneous ulcers of various etiologies has been substantiated by numerous studies (Tovey, 1991).

Manuka (*Leptospermum scoparium* J.R. Forst. and G. Forst.) is a tree native to New Zealand and southeastern Australia, belonging to the myrtle family, Myrtaceae. It is a complex natural product composed primarily of sugars, water, organic acids, enzymes, amino acids, and phenolic compounds, with a unique antimicrobial profile that distinguishes it from other honeys. Its non-peroxide antibacterial activity is largely attributed to the presence of methylglyoxal (MGO), a compound derived from dihydroxyacetone in Manuka nectar, whose concentration is quantified as the MGO value and reflects the honey’s antibacterial potency. The application of Manuka honey to wounds has been associated with reduced healing time, diminished wound size, and enhanced tissue regeneration (Al-Waili et al., 2011; Jull et al., 2015; Tashkandi, 2021). Notably, Manuka honey dressings are suitable for both dry and exudative wounds and retain antimicrobial activity even in the presence of wound exudate. These dressings are often utilized as alternatives to contemporary antimicrobial dressings containing ionic silver, particularly in cases of critical colonization, where bacterial proliferation surpasses the host’s immune response (Davies, 2005; White, 2016; Bezerra et al., 2023). A prominent example of such a condition is the diabetic foot ulcer (DFU). Evidence suggests that honey represents a cost-effective and safe therapeutic approach for DFU management, contributing to accelerated wound healing and reduced rates of amputation and hospitalization (Kamaratos et al., 2014; Tsang et al., 2015; Teobaldi et al., 2018; Bezerra et al., 2023).

**FIGURE 1:**
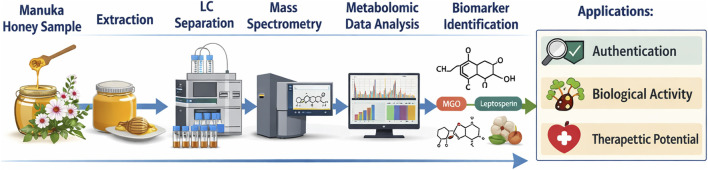
Key steps in honey authentication for clinical use.

From an authentication and mechanistic perspective, Manuka honey is commonly characterized in the literature using chemical markers such as MGO and leptosperin, alongside phenolic profiles measured by chromatographic methods (Kato et al., 2012; Kato et al., 2014; Oelschlaegel et al., 2012; Burns et al., 2018; Díaz-Galiano et al., 2023). These markers are frequently used to distinguish Manuka from non-Manuka honeys and to relate antibacterial potency to composition (Table 1). Importantly, in the present clinical trial we did not perform independent omics-scale chemical profiling, e.g.,.liquid chromatography coupled with mass spectrometry (LC–MS) of the product. Instead, we ensured clinical traceability by reporting medical device identifiers and relying on manufacturer-controlled standardization and published compositional literature. The important steps in the honey authentication procedure are shown in Figure 1.

**TABLE 1:** Most important biomarkers of Manuka honey identified by large-scale omics techniques.

Biomarker	Chemical class	Biological/analytical significance	Key references
Methylglyoxal (MGO)	Reactive dicarbonyl compound	Major antibacterial compound responsible for non-peroxide activity of Manuka honey	Mavric et al. (2008), Adams et al. (2009)
Dihydroxyacetone (DHA)	Ketone sugar	Precursor of methylglyoxal formed in Manuka nectar	Adams et al. (2009)
Leptosin (Leptosperin)	Glycoside	Phenolic marker derived from *Leptospermum scoparium* nectar	Kato et al. (2012)
Methoxyphenyllactic acid	Phenolic acid derivative	Marker associated with Manuka honey botanical origin	Oelschlaegel et al. (2012)
2-Methoxyaceto-phenone	Aromatic ketone	Volatile compound linked to *Leptospermum* species	Beitlich et al. (2014)
Syringic acid	Phenolic acid	Antioxidant compound contributing to biological activity	Oelschlaegel et al. (2012), Kato et al. (2014)
Gallic acid	Phenolic acid	Antioxidant activity and anti-inflammatory effects	Díaz-Galiano et al. (2023)

However, the use of Manuka honey in modern wound care is still met with some skepticism. Since the advent of evidence-based medicine, changing clinical practice depends on providing clinicians with appropriate levels of evidence of clinical efficacy. Although Manuka honey has become a first-line intervention in some wound care clinics, larger and better-designed randomized controlled trials are needed to cement the role of honey in modern wound care (Kapoor and Yadav, 2021).

Similar to Manuka honey, silver-containing dressings exhibit wide-ranging bactericidal activity and can substantially reduce biofilm burden *in vitro* and *ex vivo*, making them effective topical agents for infected or colonized wounds (Kostenko et al., 2010). However, important mechanistic differences justify a head-to-head comparison: Manuka honey acts via multiple physicochemical and chemically distinct pathways, whereas silver exerts its effect through ionic silver species with documented mechanisms of microbial tolerance and an increasing number of reports describing silver resistance or reduced susceptibility in wound isolates (Percival et al., 2005; Bouzo et al., 2020). It justifies direct comparison of Manuka honey vs. silver dressings in clinical wounds to determine whether differences in antimicrobial spectrum, anti-biofilm efficacy, and resistance risk translate into distinct healing outcomes (Lu et al., 2019).

Several comparative clinical and systematic studies have evaluated these two topical agents, showing that honey dressings achieve wound sterilization and healing rates comparable to or superior to silver formulations in burns and chronic wounds (Subrahmanyam, 1998; Malik et al., 2010; Lindberg et al., 2015; Aziz and Abdul Rasool Hassan, 2017; Osman et al., 2022; Kucharzewski et al., 2025). These differences in antimicrobial spectrum, biofilm disruption, and potential for resistance development provide an additional strong rationale for direct comparison between Manuka honey and silver dressings in the present study.

Importantly, among numerous clinical trials describing the positive or neutral effect of medicinal honey dressings on the treatment of DFUs (Hammouri, 2004; Shukrimi et al., 2008; Jan et al., 2012; Al Saeed, 2013; Kamaratos et al., 2014; Surahio et al., 2014; Agarwal et al., 2015; Imran et al., 2015; Tsang et al., 2015; Saeed, 2019; Bezerra et al., 2023; Holubová et al., 2023), only two (Tsang et al., 2017; Saeed, 2019) compare medicinal honey dressings to silver-based ones. The remaining studies compare honey with other substances or methods (e.g., povidone iodine, conventional treatment, no treatment, standard care) but do not include a silver dressing group. Moreover, neither of these two articles uses the precise RESVECH 2.0 scale for comprehensive healing assessment. Instead, only single parameters are assessed. Therefore, the present study fills this knowledge gap and is quite unique, because there are so far no studies that have both (1) compared honey with silver dressings in the treatment of diabetic foot ulcers and (2) used the RESVECH scale (RESVECH-2.0) to thoroughly assess the healing process. The RESVECH 2.0 (Results of the Evaluation of Chronic Wounds) scoring table is a validated wound-assessment instrument designed to quantify multiple dimensions of chronic wound status and healing over time. It evaluates six domains—wound dimensions, depth and tissues involved, edges, type of tissue in the wound bed, exudate amount, and infection/inflammation (including signs compatible with biofilm)—and produces a composite score ranging from 0 (healed) to 35 (worst condition), with lower scores indicating better healing (Cruz et al., 2023; Menegon et al., 2023).

## 2 Materials and methods

### 2.1 Research group

The randomized controlled trial included 60 patients with diabetic foot ulcers, aged from 45 to 75 (mean age 61,30 ± 7.84 years). Patients were randomly assigned to an experimental or control group. In the experimental group, patients were treated with Manuka honey, while in the control group, a silver-containing calcium alginate dressing was used. Allocation was conducted by a sealed-envelope randomization procedure. Each participant selected an opaque, sealed envelope specifying assignment to either the study or control group, ensuring participant blinding to group allocation.

**FIGURE 2:**
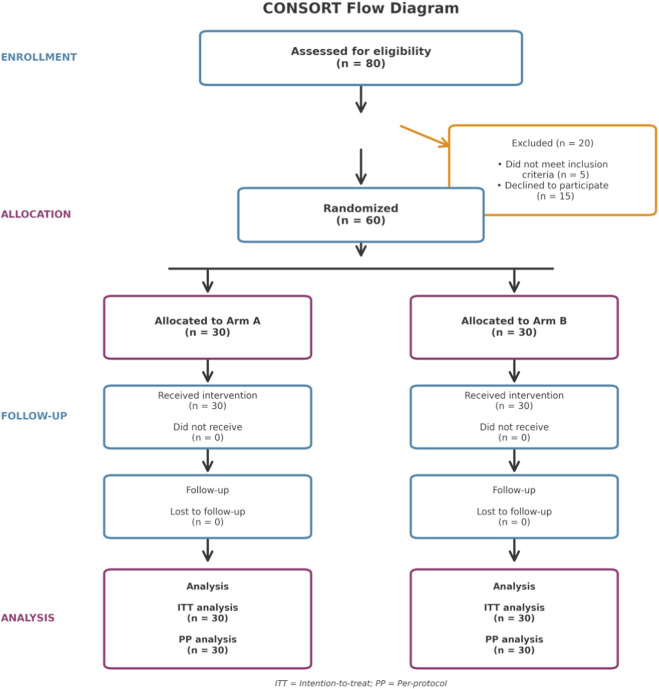
CONSORT flow diagram of participant recruitment, allocation, follow-up, and analysis. Eighty patients were assessed for eligibility; twenty were excluded (five did not meet the inclusion criteria and fifteen declined participation). Sixty participants were randomized equally into two study arms (Arm A and Arm B, *n* = 30 each). All participants received the assigned intervention and completed follow-up. No losses to follow-up occurred, and all participants were included in both intention-to-treat (ITT) and per-protocol (PP) analyses.

A total of 80 patients were screened for eligibility according to predefined inclusion and exclusion criteria. Twenty patients were excluded—five for not meeting the inclusion criteria and fifteen who declined participation. The remaining 60 participants were randomized in a 1:1 ratio into two groups: Arm A (*n* = 30) and Arm B (*n* = 30). All randomized participants received the allocated intervention and completed the scheduled follow-up. No participants were lost to follow-up, and data from all 60 subjects were included in both the intention-to-treat and per-protocol analyses. The overall study flow is summarized in Figure 2.

The study was conducted at two medical centers: Surgical Outpatient Clinic and Healthcare Centre of Jan Paweł II District Hospital in Włoszczowa and Wound Treatment Clinic of Specialist Hospital of Priest B. Markiewicz, Subcarpathian oncology Centre in Brzozowo. The data were collected from January to November 2025. All patients agreed to participate in the study and signed informed consent to confirm it. The study was approved by the Ethics Committee of Jan Długosz University in Częstochowa (protocol code KB-U/2/2025) and was conducted in accordance with the Declaration of Helsinki.

#### 2.1.1 Inclusion and exclusion criteria

Inclusion Criteria:

- A wound that has been treated unsuccessfully for more than 3 months;
- A wound with an area ranging from 10 to 40 cm^2^;
- Provided written informed consent to participate in the study
- Random glycemia not exceeding 180 mg/dL (10.0 mmol/L).

Exclusion Criteria:

- Allergy to silver or Manuka honey;
- Patients who did not provide informed consent.

#### 2.1.2 Anthropometric data

29 women (48.3%) and 31 men (51.7%) participated in the study. The number of women and men was similar in the experimental and control groups, with no statistically significant differences (*p* = 0.438). The mean age of the respondents was 61.32 ± 7.23 years, ranging from 45 to 75 years. There were no statistical differences in the age of patients in the two groups studied (*p* = 0.985). The wound area also showed no statistically significant difference (*p* = 0.700) and ranged from 10.2 to 39.1 cm^2^. Wounding time ranged from 3 to 12 months and did not differ between the two groups (*p* = 0.915). Similarly, there was no difference in the BMI (*p* = 0.964), which ranged from 23 to 40 kg/m^2^ (Table 2).

**TABLE 2:** Basic anthropometric and clinical features of patients.

Parameter	Experimental group *n* = 30	Control group *n* = 30	Sum (both groups) *n* = 60	Significance
Gender: Females Males	​	​	​	​
	13 (43.3%)	16 (53.3%)	29 (48.3%)	0.438
	17 (56.7%)	14 (46.7%)	31 (51.7%)	​
Age [years]	61.30 ± 7.84	61.33 ± 6.7	61.32 ± 7.23	t = −0.02
	(45–75)	(45–74)	(45–75)	*p* = 0.985
Wound area [cm^2^] from 10 cm^2^ to 40 cm^2^
	19.15 ± 7.8	18.94 ± 7.27	19.05 ± 7.47	Z = 0.38
	(10.2–39.1)	(12.4–35.7)	(10.2–39.1)	*p* = 0.700
Wounding time [months]	8.53 ± 2.5	8.6 ± 2.33	8.57 ± 2.4	T = −0.11
	(3–12)	(3–12)	(3–12)	*p* = 0.915
Body Mass Index (BMI) [kg/m^2^]	28.53 ± 4.07	28.47 ± 4.07	28.5 ± 4.03	Z = 0.04
	(23–40)	(23–40)	(23–40)	*p* = 0.964

t—Student’s *t*-test value; Z—Wilcoxon signed-rank test value; *p* - test probability index (*p*-value). Parameters are presented as the number of patients (percentage) or the mean value ±SD (range).

### 2.2 Research tool–RESVECH 2.0

The effectiveness of the treatment was assessed using the RESVECH (Resultados Esperados de la Valoración y Evolución de la Cicatrización de las Heridas Crónicas, eng. Results of the Evaluation of Chronic Wounds) 2.0 Scoring Table (Jais and Pratama, 2023). The scale assesses six main aspects, each contributing to a total score ranging from 35 (most severe condition) to 0 (completely healed wound). The lower the numerical result, the better the condition of the wound. The assessed parameters included: wound size, depth/tissues involved, wound edges, tissue in the wound bed, exudate and inflammation signs.

The cumulative score from all six dimensions provides an overall assessment of the wound’s condition, aiding clinicians in monitoring healing progression and tailoring treatment strategies (Jais and Pratama, 2023).

Below is a detailed breakdown of the scoring criteria for each feature:

1. Wound Size (Area): 0 points: Healed (no wound), 1 point: <4 cm^2^; 2 points: 4–16 cm^2^; 3 points: 16–36 cm^2^; 4 points: 36–64 cm^2^; 5 points: ≥64 cm^2^.
2. Depth/Tissues Involved: 0 points: Skin intact; 1 point: Involvement of epidermis and dermis; 2 points: Involvement of subcutaneous tissue; 3 points: Exposure of muscle, tendon, or bone.
3. Wound Edges: 0 points: Well-defined, attached edges. 1 point: Diffuse or indistinct edges; 2 points: Detached or undermined edges; 3 points: Thickened, rolled, or hyperkeratotic edges.
4. Tissue in the Wound Bed: 0 points: 100% epithelial tissue; 1 point: ≥75% granulation tissue; 2 points: 25%–74% granulation tissue; 3 points: <25% granulation tissue; 4 points: Presence of slough or necrotic tissue.
5. Exudate: 0 points: None; 1 point: Scant; 2 points: Moderate; 3 points: Heavy; 4 points: Copious or leaking.
6. Infection/Inflammation Signs: Each of the following signs is scored as 1 point if present, 0 if absent. The total for this section can range from 0 to 14 points: increasing pain, perilesional erythema, perilesional edema, increased local temperature, increased exudate, purulent exudate, friable tissue that bleeds easily, stagnant wound (no progress), tissue compatible with biofilm, odor, hypergranulation, increase in wound size, satellite lesions, pale tissue.

RESVECH 2.0 was developed as a revision of earlier RESVECH versions to improve clinimetric performance and has demonstrated good reliability and usefulness for monitoring chronic wound progression in clinical studies and cross-cultural adaptations. Its multidimensional approach makes it particularly useful in trials that aim to capture not only surface area reduction but also tissue quality, exudation and infection-related changes (Cruz et al., 2023; Menegon et al., 2023).

The scale was used twice–just before the treatment and again after 4 weeks.

### 2.3 Research procedure–wound care

All patients received antibiotic therapy, with the specific agent selected based on the results of the wound exudate microbiological culture. The most commonly used antibiotics were amoxicillin/clavulanate and cefuroxime. Blood glucose levels were monitored four times daily, and in all cases remained below 180 mg/dL (10.0 mmol/L).

During dressing changes, patients were positioned on an examination table in a semi-upright or semi-seated position in a room maintained at 23 °C with 40% humidity. In both groups, the wound was cleaned using an antiseptic solution containing 0.1% polyhexanide and poloxamer 188, followed by mechanical debridement with a sterile Volkmann spoon.

In the experimental group, medical-grade Manuka honey (“Activon®Tube 100% Medical Grade Manuka Honey”, Advancis Medical, Kirkby in Ashfield, Nottinghamshire, United Kingdom, batch number:W0033562, Expiration date:01.2026) was used. It is classified as a medical device intended for topical wound care and carefully selected for MGO-associated antimicrobial activity. It contains ≥95% medical-grade Manuka honey and sterile non-adherent dressing substrate. The product is characterized by standardized antibacterial activity resulting from the content of methylglyoxal (MGO), which is a quality marker of Manuka honey, and its physicochemical and microbiological parameters are controlled by the manufacturer in accordance with the requirements for medical devices. In the control group, a calcium alginate dressing with silver (Suprasorb® A+ Ag, Lohmann and Rausche, Pabianice, Poland) was applied. The product is a medical device containing approximately 1.5% ionic silver in the form of silver alginate fibers incorporated within the dressing. 10 cm^2^ dressing may release approximately 5.5 ppm of silver ions within 24 h. In both groups, the dressing was changed every 3 days.

### 2.4 Statistical analysis

Statistical analysis of the collected material was performed using StatSoft’s Statistica 13.3 package.

Nonparametric tests were used to analyze variables. The choice of a nonparametric test was determined by the failure to meet the basic assumptions of the parametric test, i.e., the distribution of the studied variables to conform to the normal distribution, which was verified using the Shapiro–Wilk W test.

Descriptive statistics were calculated for all variables: mean, median, minimum, maximum, upper and lower quartiles, and standard deviation.

Since the Shapiro–Wilk W test excluded the normal distribution, nonparametric tests were used. The Mann–Whitney U test was used to assess differences in the average level of a numerical characteristic in two populations. The Wilcoxon signed-rank test was applied to assess intragroup variability within the same population over time.

Correlation between two variables that did not meet the criterion of normality was determined using the Spearman rank correlation coefficient. Analysis of qualitative variables was performed using the Pearson chi-square test. The level of statistical significance was set at *p* < 0.05.

## 3 Results

**FIGURE 3:**
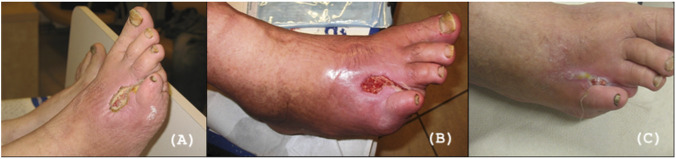
Ulcer healing process (during Manuka honey treatment) of 60-year-old woman with a DFU. **(A)** Initial presentation of the ulcer on the right foot. **(B)** Clinical appearance of the wound after 14 days of treatment with Manuka honey gel, showing a reduction in inflammation and partial development of granulation tissue. **(C)** Clinical appearance after 21 days of treatment, demonstrating further wound healing and improved granulation tissue formation.

**FIGURE 4:**
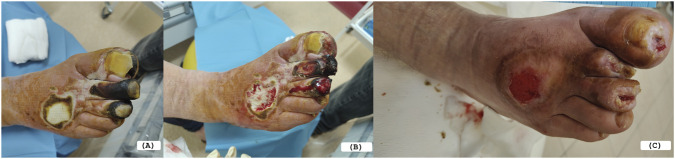
Clinical progression of ischemic DFUs of 68-year-old man treated with Manuka honey. **(A)** Status after amputation of the distal phalanx of the second toe of the right foot with necrosis of the distal pulp of the first and third toes. **(B)** Wound appearance after 3 days of treatment with medical-grade Manuka honey, showing clear demarcation of necrotic tissue. **(C)** Wound status after 21 days of treatment, demonstrating progressive granulation tissue formation and wound healing.

Figures 3, 4 show examples of the treatment effect in the experimental group at three time points. The first one concerns 60-year-old female patient with a 20-year history of type 2 diabetes mellitus. Eight years prior to admission, the patient had undergone amputation of the fourth toe of the right foot due to complications of diabetic foot syndrome. The patient reported an ulcer on the right foot that had not healed for 30 days. Clinical examination revealed an ulcerative lesion with signs of local inflammation, with the presence of fibrin deposits and granulation tissue. Topical treatment with Manuka honey gel was initiated and during follow-up, a gradual improvement in the wound appearance was observed, with reduction of inflammatory signs and progressive development of healthy granulation tissue, leading to visible wound healing (Figure 3). The second example is a 68-year-old male with a 15-year history of type 2 diabetes mellitus and peripheral artery disease (PAD) who was admitted for treatment of ischemic diabetic foot complications. Two months prior to admission, the patient underwent revascularization of the arteries of the right lower limb due to critical limb ischemia. One month later, amputation of the distal phalanx of the second toe of the right foot was performed because of progressive ischemic necrosis. At the same time, dry necrosis of the distal pulp of the first and third toes was observed and managed conservatively with demarcation. After 3 days of Manuka honey treatment, clear demarcation of necrotic tissues was observed with reduction of inflammatory signs and improved wound bed condition. After 21 days of treatment with Manuka honey, progressive wound healing was observed with formation of healthy granulation tissue and gradual epithelialization of the wound margins (Figure 4).

### 3.1 Absolute scores on the REVESTECH 2.0 scale

The exact effectiveness of the treatment was assessed based on the scores (in points) on the RESVECH 2.0 scale within 4 weeks of treatment beginning.

#### 3.1.1 Experimental group

Statistically significant differences between the initial and final values were demonstrated in the experimental group for the following parameters: Wound Size (Area), Wound Edges, Tissue in the Wound Bed, Exudate, signs of infection or inflammation, and the total score (*p* < 0.001). In each of the abovementioned categories, the results after 4 weeks of treatment were more favorable compared to the initial measurement, i.e., after 1 month of treatment, the RESVECH 2.0 scale scores were significantly lower. The most spectacular improvement (over 50%) was observed for exudate and signs of infection or inflammation. The overall score was significantly reduced by 41.3% (Table 3).

**TABLE 3:** Wound treatment effectiveness (points in RESVECH 2.0 scale)–experimental group.

Experimental group *n* = 30		Mean	Median	Min	Max	Quartile I	Quartile III	SD
WoundSize (Area)	Before	2.6	3.0	2.0	4.0	2.0	3.0	0.62
	4 weeks after	1.7	2.0	0.0	4.0	1.0	2.0	0.88
Z = 4.45 *p* < 0.001, d = 1.45, **reduction by 34.6%**								
Depth/tissues involved	Before	2.2	2.0	2.0	3.0	2.0	2.0	0.38
	4 weeks after	2.1	2.0	0.0	3.0	2.0	2.0	0.58
Z = 1.34 *p* = 0.179, d = 0.26, **reduction by 4.5%**								
Wound edges	Before	2.7	3.0	2.0	3.0	2.0	3.0	0.48
	4 weeks after	1.7	2.0	0.0	2.0	1.0	2.0	0.53
Z = 4.54 *p* < 0.001, d = 2.02, **reduction by 37.0%**								
Tissue in the wound bed	Before	3.6	4.0	3.0	4.0	3.0	4.0	0.49
	4 weeks after	2.1	2.0	0.0	3.0	2.0	2.0	0.52
Z = 4.78 p < 0.001, d = 3.18, **reduction by 41.7%**								
Exudate	Before	1.8	2.0	1.0	4.0	1.0	2.0	0.95
	4 weeks after	0.8	1.0	0.0	3.0	0.0	1.0	0.79
Z = 4.54 *p* < 0.001; d = 1.05, **reduction by 55.6%**								
Infection/inflammation signs	Before	7.2	7.0	6.0	9.0	6.0	8.0	1.02
	4 weeks after	3.5	3.0	0.0	6.0	3.0	4.0	1.25
Z = 4.78 p < 0.001, d = 3.63, **reduction by 51.4%**								
Sum of points (total score)	Before	20.1	19.5	16.0	27.0	17.0	22.0	3.34
	4 weeks after	11.8	12.0	0.0	20.0	10.0	13.0	3.74
Z = 4.78 *p* < 0.001, d = 2.47, **total score reduction by 41.3%**								

Mi, minimum value; Max, maximum value; SD, standard deviation; Z, Wilcoxon signed-rank test value; p, test probability index (*p*-value); d, effect size; the percentage reduction/increase in the number of points on the RESVECH, scale refers to the mean values.

Reductions in points in RESVECH 2.0 scale are showed in bold.

#### 3.1.2 Control group

In the control group, significant differences between the initial and final measurements were demonstrated for the following parameters: Tissue in the Wound Bed (*p* = 0.017) and Exudate (*p* = 0.027). In the case of Tissue in the Wound Bed, the parameters improved after 4 weeks, as indicated by a lower numerical score, while in the case of Exudate, they worsened. Other wound parameters did not change at all. The overall score was reduced only by 2% (Table 4).

**TABLE 4:** Wound treatment effectiveness (points in RESVECH 2.0 scale) – control group.

Control group *n* = 30		Mean	Median	Min	Max	Quartile I	Quartile III	SD
Wound size (Area)	Before	2.5	2.5	2.0	3.0	2.0	3.0	0.51
	4 weeks after	2.4	2.5	1.0	3.0	2.0	3.0	0.63
Z = 1.34 *p* = 0.179, d = 0.20, **reduction by 4.0%**								
Depth/tissues involved	Before	2.2	2.0	2.0	3.0	2.0	2.0	0.38
	4 weeks after	2.2	2.0	2.0	3.0	2.0	2.0	0.38
-								
Wound edges	Before	2.6	3.0	2.0	3.0	2.0	3.0	0.50
	4 weeks after	2.6	3.0	2.0	3.0	2.0	3.0	0.50
-								
Tissue in the wound bed	Before	3.6	4.0	3.0	4.0	3.0	4.0	0.49
	4 weeks after	3.4	3.0	3.0	4.0	3.0	4.0	0.50
Z = 2.36 *p* = 0.017, d = 0.41, **reduction by 5.6%**								
Exudate	Before	1.77	2.0	1.0	3.0	1.0	2.0	0.77
	4 weeks after	1.97	2.0	1.0	4.0	1.0	3.0	0.96
Z = 2.20 *p* = 0.027, d = 0,26, **increase by 11.3%**								
Infection/inflammation signs	Before	7.1	7.0	6.0	9.0	6.0	8.0	1.11
	4 weeks after	6.8	6.5	5.0	10.0	6.0	8.0	1.19
Z = 1.65 *p* = 0.097, d = 0.27, **reduction by 4.2%**								
Sum of points (total score)	Before	19.7	19.5	16.0	25.0	17.0	22.0	3.25
	4 weeks after	19.3	19.0	14.0	27.0	17.0	22.0	3.35
Z = 1.45 *p* = 0.144, d = 0.12, **total score reduction by 2.0%**								

Reductions in points in RESVECH 2.0 scale are showed in bold.

### 3.2 Changes in scores on the REVESTECH 2.0 scale after 4-week-Long treatment

**FIGURE 5:**
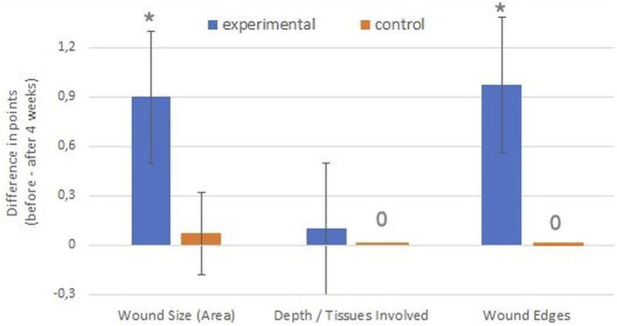
Comparison of treatment effects in two groups regarding anatomical wound features–differences in the numerical scores (points in RESVECH 2.0 scale) before treatment and 4 weeks after treatment. *p < 0.001 vs. control group.

**FIGURE 6:**
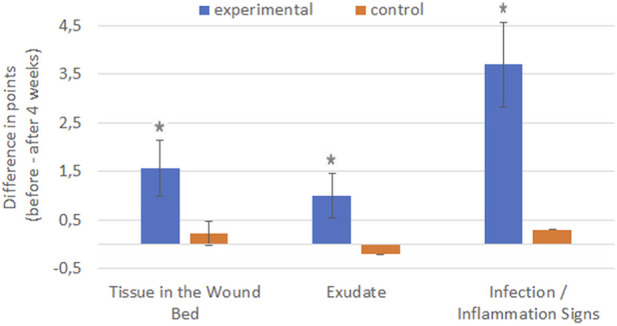
Comparison of treatment effects in two groups regarding functional wound features–differences in the numerical scores (points in RESVECH 2.0 scale) before treatment and 4 weeks after treatment. *p < 0.001 vs. control group.

The treatment effects achieved in the experimental and control groups over the 4-week treatment period were compared. The treatment effect for almost all parameters (except Depth/Tissues Involved) was greater in the experimental group than in the control group (*p* < 0.001), as indicated by significantly greater differences in the RESVECH 2.0 scores obtained at these two time points (Figures 5, 6).

**FIGURE 7:**
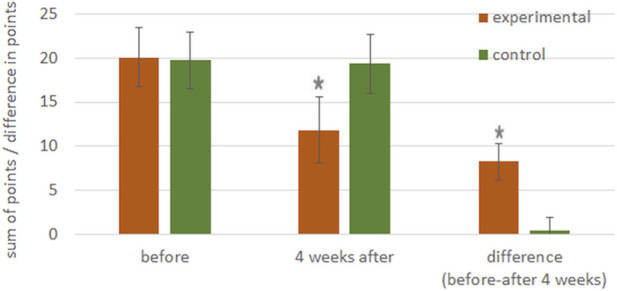
Total scores (sum of points in RESVECH 2.0 scale) for the experimental and control group and the difference between them were calculated as total score before treatment minus total score 4 weeks after treatment. *p < 0.001 vs. score before treatment (for sum of points), *p < 0.001 vs. control group (for difference in points).

Total score (sum of points on RESVECH 2.0 scale) did not change in the control group, whereas in experimental group it was significantly lower 4 weeks after treatment, than at the beginning of the study. The absolute value of this difference was also high enough to reach statistical significance vs. the control group (Figure 7).

**FIGURE 8:**
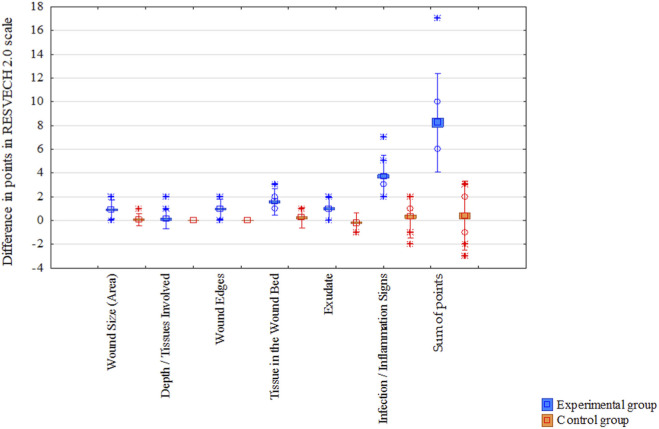
Comparison of treatment effects in two groups–point differences in RESVECH 2.0 scale before treatment and 4 weeks after treatment. *p < 0.001 vs. control group.

To sum up, the mean RESVECH 2.0 scores decreased over time in both groups, indicating progressive wound healing. However, the reduction was more pronounced in the Manuka honey group compared to the silver dressing group. By the end of the observation period, wounds treated with Manuka honey demonstrated significantly better healing outcomes, as reflected by lower RESVECH 2.0 scores (Figure 8).

### 3.3 Correlations between scores on the REVESTECH 2.0 scale and wounding time

Statistically significant correlations between wounding time and RESVECH 2.0 scale scores before and after 4 weeks of treatment are marked in bold, as well as between wounding time and the treatment effect (the difference between pre- and post-treatment scores). Almost all of these correlations were positive. This means that the longer the patient suffered from the wound, the higher (worse) their scores were both before and after treatment. As the wounding time increased, the treatment effect also improved, especially in the experimental group for parameters such as Tissue in the Wound Bed and Exudate (Table 5).

**TABLE 5:** Points in RESVECH 2.0 scale vs. wounding time - correlations.

Experimental group	Before treatment		4 weeks after treatment		Difference (effect)	
	R	*p*	R	*p*	R	*p*
Wound size (Area)	0.85	<0.001	0.76	<0.001	−0.20	0.299
Depth/tissues involved	0.51	0.004	0.50	0.005	−0.17	0.364
Wound edges	0.78	<0.001	0.56	0.001	0.24	0.197
Tissue in the wound bed	0.58	0.001	0.10	0.598	0.43	0.017
Exudate	0.78	<0.001	0.60	<0.001	0.48	0.007
Infection/inflammation signs	0.63	<0.001	0.50	0.005	0.20	0.297
Sum of points	0.85	<0.001	0.72	<0.001	0.44	0.014

Control group	Before treatment		4 weeks after treatment		Difference (effect)	
	R	*p*	R	*p*	R	*p*
Wound Size (Area)	0.81	<0.001	0.74	<0.001	−0.03	0.870
Depth/Tissues Involved	0.59	0.001	0.59	0.001	-	-
Wound Edges	0.82	<0.001	0.82	<0.001	-	-
Tissue in the Wound Bed	0.68	<0.001	0.34	0.064	0.38	0.040
Exudate	0.88	<0.001	0.86	<0.001	−0.32	0.088
Infection/Inflammation Signs	0.74	<0.001	0.60	<0.001	0.19	0.320
Sum of points	0.89	<0.001	0.84	<0.001	0.11	0.558

R—Spearman’s rank correlation test value; p—test probability index (*p*-value).

## 4 Discussion

An optimal wound dressing would ideally possess broad-spectrum antimicrobial properties, exhibit biocompatibility with uninjured tissue, and maintain its effectiveness over an extended duration (Khansa et al., 2019). Despite the widespread use of various topical dressings to aid DFU healing, there is still insufficient high-quality evidence to support standardized clinical decision-making (Bergin and Wraight, 2006; Jull et al., 2015). Although systematic reviews suggest that certain alternative dressings may accelerate wound healing in comparison to silver sulfadiazine, honey-based dressings appear to offer more consistent advantages in reducing wound infection (Aziz and Abdul Rasool Hassan, 2017; Rashaan et al., 2019).

Recently, there has been a renewed scientific interest in honey application on hard-to-heal wounds, supported by a growing body of evidence from animal models, clinical studies, case reports, and randomized controlled trials demonstrating its comparable efficacy to contemporary wound dressing materials in promoting wound healing (Vit and HuQ, 2008).

Recent studies have provided deeper insight into the molecular mechanisms underlying honey’s pro-healing activity. One of the key effects involves modulation of immune responses, as honey stimulates leukocytes to release cytokines and growth factors that enhance keratinocyte expression of TNF-α, IL-1β, and TGF-β, thereby promoting tissue repair (Majtan et al., 2013; Jull et al., 2015). In addition to these immunomodulatory effects, honey induces calcium influx into keratinocytes through hydrogen peroxide–mediated activation of glucose oxidase, a process that facilitates wound closure (Martinotti et al., 2019; 2023). Anti-inflammatory actions have also been linked to the inhibition of the NF-κB signaling pathway, leading to reduced expression of inflammatory mediators such as COX-1 and COX-2 (Scepankova et al., 2021; Bezerra et al., 2023). Furthermore, honey’s naturally low pH creates an acidic microenvironment unfavorable to microbial growth while simultaneously enhancing oxygen release from hemoglobin, stimulating angiogenesis, cytokine production, and granulation tissue formation (Scepankova et al., 2021; Bezerra et al., 2023).

Our study confirms the abovementioned promising potential of honey as an alternative to traditional diabetic foot treatment, even in the case of long-term wound healing, as evidenced by the correlation between the RESVECH 2.0 score and wounding time.

Medical-grade Manuka honey (100%) used in the study was in sterile, preservative-free gel form (Activon® Tube, Advancis Medical, Nottingham United Kingdom). The viscous formulation allows direct application or use with honey-impregnated carriers (mesh, alginate, hydrogel, or foam). Honey’s high viscosity and low water activity enable retention at the wound site while gradual dilution by exudate produces an initial burst and subsequent diffusion-controlled release of methylglyoxal (MGO), hydrogen-peroxide precursors, and organic acids that maintain an acidic microenvironment. Release rate depends on temperature-related viscosity, exudate volume, and dressing absorbency (Molan and Rhodes, 2015).

Manuka honey is biocompatible and non-cytotoxic, supporting autolytic debridement and granulation, with allergy to bee products being the only contraindication (Seckam and Cooper, 2013). Bioactive stability (MGO, peroxide system) is maintained under cool, light-protected storage; excessive heat or dilution reduces activity and may cause crystallization, mitigated by controlled moisture and filtration (Kwakman et al., 2010).

Because steam sterilization degrades Manuka honey components, sterility is ensured *via* aseptic processing and terminal γ-irradiation, validated to inactivate bacterial spores while preserving bioactivity. Packaging enables single-patient use and minimizes tip contamination; opened products are used within the labeled in-use period under an occlusive secondary dressing (Gościniak et al., 2025; Peters et al., 2025).

**FIGURE 9:**
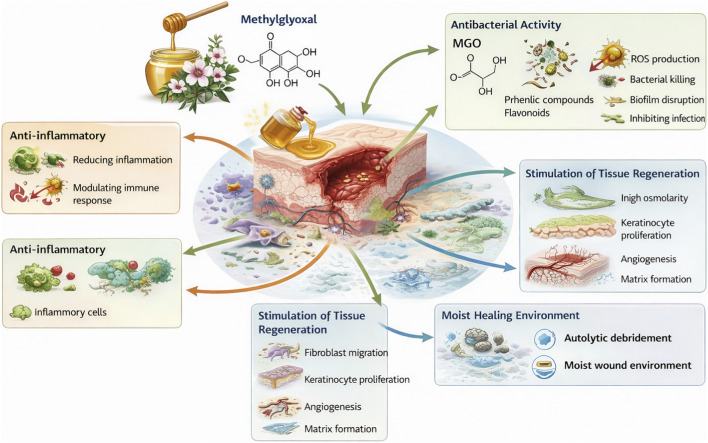
Main mechanisms of therapeutic effect of Manuka honey.

To sum up, although present study was clinical and did not include omics-scale chemical verification (e.g., LC–MS profiling) of the product, the most plausible drivers of the observed clinical benefit are consistent with well-described Manuka honey features reported in the literature: 1) non-peroxide antibacterial activity associated with methylglyoxal (MGO) and related carbonyl chemistry, 2) an acidic, high-osmolar microenvironment that suppresses bacterial growth and supports oxygen release and granulation, and 3) a phenolic fraction with antioxidant/anti-inflammatory effects that may modulate excessive chronic inflammation typical for DFUs. In practical wound settings, these concurrent mechanisms may translate into reduced local inflammatory burden, decreased exudate, and improved wound bed tissue quality—exactly the domains where RESVECH 2.0 differences were most pronounced in our cohort. Main factors likely responsible for the demonstrated therapeutic effect of Manuka honey are presented in Figure 9. These properties have a direct impact on the antibacterial effect and the resulting clinical effectiveness of Manuka honey, which we have also demonstrated in our study (Johnston et al., 2018; Girma et al., 2019; Nolan et al., 2020).

The precise biological pathways responsible for impaired wound healing in diabetic conditions remain incompletely understood (Blakytny and Jude, 2006). Loots et al. (1999) demonstrated that fibroblasts derived from diabetic wounds exhibit reduced proliferative potential and atypical morphological features. Similarly, Galkowska et al. (2005) in an *in vitro* investigation, suggested that DFU healing may be compromised by impaired leukocyte accumulation. In a chemotaxis assay involving monocytes isolated from individuals with diabetes, Waltenberger et al. (2000) reported a diminished responsiveness to vascular endothelial growth factor (VEGF) compared to monocytes from non-diabetic controls. Immunohistochemical analysis by Usui et al. (2008) revealed that keratinocyte migration and differentiation are significantly disrupted at the margins of chronic ulcers in diabetic patients. Furthermore, Albiero et al. (2011) identified that delayed wound healing in diabetic mice is linked to defective recruitment, survival, and proliferation of bone marrow–derived endothelial progenitor cells. Additionally, macrophages from diabetic mice show a predominance of pro-inflammatory M1 phenotypes, which are believed to exacerbate wound healing deficits in the diabetic context (Usui et al., 2008). Collectively, these abnormalities illustrate how diabetes induces a multifactorial impairment of wound healing that underlies the chronic nature of DFUs (Blakytny and Jude, 2006).

Taking into account the abovementioned complex nature of wound healing problems in the course of diabetes, a precise wound assessment scale (RESVECH 2.0) was applied, which assessed not only the size and depth of the wound, but also its physiological characteristics such as the type of exudate and tissue specificity. This scale has gained popularity in recent years and is used to monitor and evaluate the healing of various types of chronic wounds (Jiménez-García et al., 2021; Comino-Sanz et al., 2023). There is also a study on monitoring the healing of diabetic foot specifically, in which the protocol used the RESVECH 2.0 scale as one of the measures of the healing process (Iruela Sánchez et al., 2023). Similarly, Sharma et al. conducted a prospective study of a new diabetic foot wound dressing, the results of which included changes in RESVECH 2.0 scores used as a measure of treatment effect (Sharma et al., 2025).

The results of the present randomized controlled trial demonstrate that the use of Manuka honey dressings leads to significantly greater improvement in the healing of DFUs compared to treatment with silver-containing calcium alginate dressings. Except for the parameter “Depth/Tissues Involved,” all evaluated aspects of the RESVECH 2.0 scale showed superior outcomes in the honey-treated group. These findings confirm the therapeutic potential of honey-based products in diabetic wound management and are consistent with previous studies highlighting their efficacy in promoting wound closure and tissue regeneration, as reviewed by Jull et al. (2015), Al-Waili et al. (2011), Yildiz Karadeniz and Kaplan Serin (2023) and Tsang et al. (2015).

However, a pilot randomized controlled trial conducted by Tsang et al. (2017) did not show such clear results as ours. Three types of dressings were compared: nanocrystalline silver, Manuka honey, and conventional dressing. While wounds treated with Manuka honey tended to show faster early healing and better reduction in wound size, these differences were not statistically significant after 12 weeks of treatment (Tsang et al., 2017). The discrepancy between our findings and those of Tsang et al. may be explained by several factors. Firstly, differences in dressing protocols could have influenced treatment efficacy—our study compared Manuka honey directly with a silver-based dressing, whereas Tsang et al. included an additional conventional dressing group, which may have diluted group comparisons. Secondly, variations in baseline ulcer characteristics, including infection severity and chronicity, could have affected healing dynamics, as Manuka honey is particularly effective in managing infected or exudative wounds. Finally, the use of different wound assessment tools may have contributed to divergent outcomes; while our study employed the RESVECH 2.0 scale (which captures multiple dimensions of wound healing), Tsang et al. primarily evaluated wound size reduction, potentially overlooking subtler improvements in tissue quality and healing progression.

Similarly, a Saudi Arabian study involving 71 patients with diabetic neuropathic plantar ulcers did not show significant differences between Manuka honey and ionic silver hydrogel efficacy. Although the mean healing time was shorter in the Manuka honey group, and the silver dressing group showed slightly faster infection control and shorter hospitalization, none of these differences reached statistical significance (Saeed, 2019). This discrepancy may be explained by methodological variations—including differences in study endpoints, dressing composition (hydrogel vs. alginate), assessment tools (clinical vs. RESVECH scoring), and sample characteristics (neuropathic vs. mixed DFU types).

The described studies and other relevant clinical studies of honey in the treatment of DFUs are summarized in Table 6. Across randomized and prospective clinical studies summarized in Table 6, Manuka honey–based dressings were associated with improved wound healing parameters in diabetic foot ulcers (DFUs), including faster reduction of ulcer size, improved granulation, and better infection control. Taken together, both presented results and most previous studies as well as review articles highlight the therapeutic superiority of medicinal honey-based dressings over silver formulations in the management of DFUs. From a clinical standpoint, these consistent findings support the consideration of medical-grade honey dressings—particularly those containing Manuka honey—as an evidence-based alternative to conventional silver dressings in DFU care.

**TABLE 6:** Clinical studies evaluating the effectiveness of Manuka honey–based dressings in the treatment of diabetic foot ulcers (DFUs), including comparisons with silver-containing dressings.

Study	Study design	Sample size	Intervention	Key outcomes
Tsang et al. (2017)	Pilot randomized controlled trial	n = 31	Three groups: nanocrystalline silver dressing, Manuka honey dressing, and conventional dressing	Both Manuka honey and nanocrystalline silver groups showed improved healing compared with conventional dressings. Honey demonstrated good infection control and promoted granulation tissue formation
Saeed (2019)	Prospective randomized clinical trial	n = 50	Controlled-release ionic silver hydrophilic dressing vs. medicated honey-impregnated dressing	Both treatments promoted wound healing. Honey dressings showed faster slough reduction and earlier granulation tissue formation compared with silver dressings
Kamaratos et al. (2014)	Prospective randomized controlled study	n = 63	Manuka honey-impregnated dressing vs. conventional saline-soaked gauze	Honey significantly shortened healing time and accelerated bacterial clearance
Imran et al. (2015)	Randomized controlled trial	n = 348	Honey dressing vs. povidone-iodine dressing	Honey treatment resulted in significantly faster healing and reduced infection rates
Moghazy et al. (2010)	Prospective clinical trial	n = 30	Honey dressing applied to DFUs	Honey promoted rapid wound cleansing, reduced edema and exudate, and improved healing rates
Nasir et al. (2010)	Randomized clinical trial	n = 150	Honey dressing vs. normal saline dressing	Higher proportion of healed ulcers and faster healing in the honey group

### 4.1 Clinical implications, limitations and future perspectives

The clinical implications of the present findings are particularly important in the context of resource-limited healthcare settings, where access to advanced wound care technologies may be restricted. Manuka honey represents a biologically active, and easily accessible therapeutic option that can provide effective wound management without the financial burden associated with synthetic or nanoengineered dressings. Recent advances in biomaterial research, such as nanozyme- or hydrogel-based systems, have demonstrated promising effects on diabetic wound healing through antimicrobial, anti-inflammatory, and antioxidant mechanisms. For example, Zhang et al. (2025) developed a spatial confinement biological heterogeneous cascade nanozyme composite hydrogel combined with nitric oxide gas therapy, achieving enhanced outcomes in diabetic wound models. Similarly, Tai et al. (2025) reported a glucose-responsive nanozyme hydrogel capable of both glycemic control and catalytic anti-infective activity, further underscoring the importance of bioactive wound environments. In addition, Liu et al. (2023) highlighted the anti-inflammatory and antioxidant effects of chrysin in mitigating diabetic foot ulcers. Collectively, these studies emphasize the growing interest in biologically inspired and multifunctional wound therapies, within which honey remains an attractive, practical, and affordable alternative—particularly valuable in low-resource settings where cost-effective solutions are crucial.

The present study has several limitations that should be acknowledged. Firstly, the relatively small sample size (60 participants) limits the generalizability of the findings and may reduce the statistical power to detect subtle differences between patient groups. Secondly, the follow-up period of 4 weeks may not have been sufficient to assess long-term outcomes, such as complete wound closure, recurrence rates, or sustained healing quality. Thirdly, the lack of blinding of participants and investigators may have introduced potential bias in treatment assessment. Moreover, variability in ulcer characteristics (e.g., size, chronicity, infection status) and patient-related factors (such as glycemic control and comorbidities) could have influenced the healing process despite randomization. Another limitation is the absence of mechanistic biomarker assessment (e.g., wound-fluid cytokines/proteases by ELISA) and the lack of independent product composition verification using analytical platforms (e.g., targeted MGO/leptosperin assays or LC–MS metabolomics. Therefore, the present work supports clinical effectiveness but cannot directly attribute outcomes to a quantified chemical profile of the investigational honey. Finally, as the study was conducted in two clinical centers, the results may not be fully representative of other healthcare settings or broader patient populations.

Future research should focus on confirming these findings in larger, multicenter randomized controlled trials with longer follow-up periods to evaluate sustained healing and recurrence prevention. Moreover, comparative studies involving different types and concentrations of medical-grade honey as well as Manuka honey with different MGO values could provide deeper insight into optimal formulations and application protocols. Additionally, investigations combining honey-based dressings with adjunctive therapies, such as negative pressure wound therapy or growth factor applications, may further enhance clinical outcomes. Similarly, combined treatment using, for example, larva therapy, Manuka honey and silver alginate dressings could be promising, as shown by a case study published by Faraji et al. (2023). An interesting idea would also be to investigate microbiological properties of wounds, especially since experimental research has demonstrated that Manuka honey induces alterations in the molecular architecture of several bacterial species, including *Staphylococcus aureus* (MRSA-15) (Cooper et al., 2002; Blair et al., 2009; Henriques et al., 2010) *Pseudomonas aeruginosa* (Henriques et al., 2011; Roberts et al., 2012), *Escherichia coli* (Blair et al., 2009), and *Streptococcus pyogenes* (Group A *streptococci*) (Cooper et al., 2002).

### 4.2 Conclusion

The results of this study suggest that Manuka honey may support improved healing outcomes in patients with DFUs compared to a standard silver-based dressing. The observed differences across several parameters indicate a possible beneficial effect of honey on tissue repair and the overall wound-healing process. These findings contribute to the growing body of evidence supporting Manuka honey as a potential adjunct in diabetic wound management. Moreover, the association between wound duration and treatment response observed in the experimental group suggests that medicinal honey-based therapy could be particularly useful in patients with chronic or hard-to-heal ulcers.

## Data Availability

The raw data supporting the conclusions of this article will be made available by the authors, without undue reservation.
